# A Cross-Sectional Study of the Relationship Between Mental Health Problems and Overweight and Obesity in Adolescents

**DOI:** 10.3389/fpubh.2020.00334

**Published:** 2020-08-18

**Authors:** Asborg Aanstad Bjertnaes, Ingrid Nesdal Fossum, Ingvild Oma, Kjersti Sletten Bakken, Tor Arne, Mads Nikolaj Holten-Andersen

**Affiliations:** ^1^Department of Pediatrics, Lillehammer Hospital, Innlandet Hospital Trust, Lillehammer, Norway; ^2^Department of Clinical Medicine, Faculty of Medicine, University of Oslo, Oslo, Norway; ^3^Division of Mental Health Care, BUP Lillehammer, Innlandet Hospital Trust, Lillehammer, Norway; ^4^Department of Medical Microbiology, Innlandet Hospital Trust, Lillehammer, Norway; ^5^Women's Clinic, Innlandet Hospital Trust, Lillehammer, Norway; ^6^Department of Research, Innlandet Hospital Trust, Brumunddal, Norway

**Keywords:** adolescence, body mass index, gender, mental health problems, obesity, overweight, SDQ

## Abstract

**Background:** There is a suggested coexistence between obesity and mental health discomfort in adolescence. The objective of this study was to explore if mental health indices covaried with body mass index (BMI) in adolescence and if there were gender-related disparities.

**Methods:** Data were collected in two cross-sectional surveys of 10th-grade students (15 to 16 years old) carried out in 2002 and 2017. The questionnaires included self-reported height and weight, questions covering mental health using the Strengths and Difficulties Questionnaire (SDQ), lifestyle, and sociodemographic variables. We estimated the associations between SDQ subscale scores and BMI and the prevalence of overweight and obesity in linear and logistic multivariable models. We also estimated the extent to which gender modified these associations.

**Results:** BMI was positively associated with peer problems [beta (β): 0.08, (95% confidence interval 0.01, 0.14)], indicating that for every point increase in peer problems subscore, BMI increased by 0.08 kg/m^2^. The association between internalizing (i.e., peer and emotional) problems and BMI and conduct problems and BMI was different for boys and girls (*p* < 0.05 for all effect modifications).

**Conclusion:** In this repeated cross-sectional study across 15 years, we found that peer problems were associated with BMI in Norwegian adolescents. We also found that there is a possibility that adolescent boys and girls report different mental health symptoms related to increased BMI. This finding implicates a need for gender-specific attention when assessing risk factors for increased BMI in adolescents.

## Introduction

The adolescent years are highly influential to health in adulthood (1), as essential capabilities related to physical and mental health and well-being develop during adolescence (2). However, unhealthy habits may also be established during this period of life (3), and global trends of unhealthy lifestyle represent a threat to adolescent health (2). Further, many common mental health problems commence during adolescence, especially in girls (4).

The adolescent disease burden has changed in most western countries during the last 25 years, with a shift from injuries and contagious diseases toward non-transmissible conditions like obesity and mental health problems (5). Studies reveal significant increases in the prevalence of both obesity and internalizing symptoms in adolescents globally (6, 7), resulting in comparable global prevalence estimates for both conditions (8).

Earlier studies have explored the possible association between mental health discomfort and increased body mass index (BMI) in adolescents. A threshold effect of BMI on mental health indices has been found (9), and the association differed between clinical and population-based samples (10). Further, the direction of the association between adolescent BMI and mental health discomfort has been found as possibly reciprocal or even lacking (11, 12). A knowledge gap regarding BMI and externalizing mental health discomfort has also been found (13, 14). For gender-related differences, suggestions have been made regarding depression and BMI (15) and externalizing problems and BMI, although with several limitations (15). In sum, the findings on the possible association between mental health and BMI in adolescence need to be further explored.

Our first aim was to investigate if mental health indices covaried with increased BMI in a population-based study of 15 to 16-year-old adolescents. We used four subscales from the Strengths and Difficulties Questionnaire (SDQ); emotional symptoms, peer problems, conduct problems, and hyperactivity as indices of mental health problems. The second aim was to examine if these associations differed between genders.

We hypothesized that for each subscale on SDQ, reports of more mental health problems would be associated with a higher BMI. Regarding gender differences, we hypothesized that BMI in girls would be affected by emotional and peer problems, and BMI in boys would be influenced by conduct and hyperactivity problems.

## Materials and Methods

### Procedure and Participants

**Figure 1:**
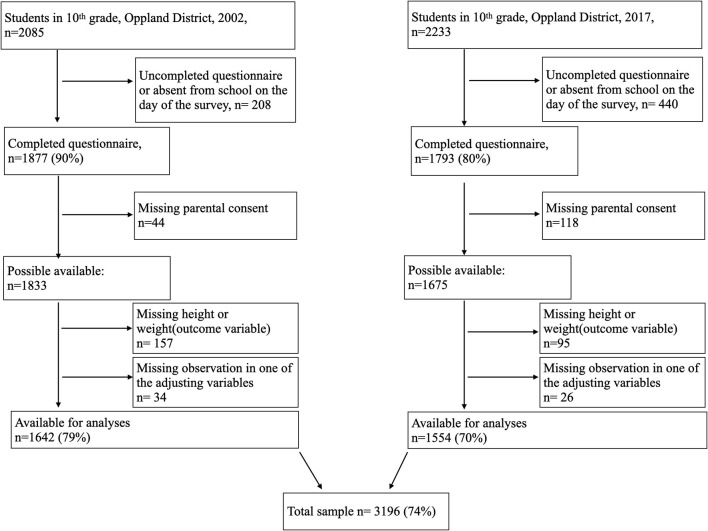
Flowchart.

Data were collected in two cross-sectional surveys among 10th-grade students (15 to 16 years old) in lower secondary schools in the district of Oppland, Norway, in 2002 and 2017. The district of Oppland had a total population of 183,000 in 2002 and 189,000 in 2017 and is predominantly a rural district with three towns with populations up to 30,000 each. The survey contained questions on mental health, nutrition, leisure-time sports, and current weight and height. In 2002, the Norwegian Institute of Public Health conducted the survey, and in 2017, our research group conducted it in collaboration with the county governor of Oppland. There were pilot studies of the questionnaires among 10th-grade students both in 2002 (16) and 2017. Participation was voluntary, and the surveys were completed during one school hour. In Norway, 10 years of education is mandatory, and thus all 15 to 16-year-old adolescents in the district were invited to the survey. The survey was done in all 46 schools in Oppland in 2002 and in all 43 public schools in 2017. Totally, 1,877 of 2,085 (90% of adolescents) completed the questionnaire 2002, and 1,788 of 2,233 (80% of adolescents) completed the questionnaire in 2017. However, due to missing consent and missing data on height or weight, the study sample comprises 3,196 participants, 1,642 in 2002, and 1,554 in 2017 (Figure 1). The mean age of the adolescents was 15.9 years in 2002 and 15.8 years in 2017. The surveys included 809 girls at both time points, which were 49.3% of the participants in 2002 and 52.1% in 2017. Table 1 displays the other adjusting variables in the two surveys.

**Table 1:** Characteristics of study participants.

	**2002 *n* = 1,642**	**2017 *n* = 1,554**
**Gender**		
Male	833 (50.7)	745 (47.9)
Female	809 (49.3)	809 (52.1)
Age; years, mean (*SD*)	15.9 (0.3)	15.8 (0.3)
Weight; kg, mean (*SD*)	62.2 (11.0)	63.5 (11.3)
Height; cm, mean (*SD*)	171.5 (8.5)	171.9 (8.6)
BMI (kg/m^2^); mean (*SD*)	21.1 (3.0)	21.4 (3.1)
BMI z-score; mean (*SD*)^a^	0.06 (1.0)	0.19 (1.0)
**Weight class^b^**		
Underweight	143(8.7)	98 (6.3)
Normal weight	1,286 (78.3)	1,196 (77.0)
Overweight	179 (10.9)	220 (14.2)
Obesity	34 (2.1)	40 (2.6)
**Adjusting variables**		
**Perceived family economy**		
Poor	58 (3.5)	62 (4.0)
Average	637 (38.8)	471 (30.3)
Good	869 (52.9)	848 (54.6)
Very good	78 (4.8)	173 (11.1)
Member of leisure-time sports team	714 (43.5)	882 (56.8)
Eating daily breakfast	1,086 (66.1)	978 (62.9)

*N = 3,196*.

*Data are presented as n (%) unless indicated otherwise*.

a *BMI adjusted for age and gender*.

b *According to International Obesity Task Force (IOTF)*.

*BMI, body mass index*.

### Outcome Variables

Planning for linear and logistic regression analyses, we calculated two outcome variables based on the self-reported anthropometric data weight to the nearest kilogram and height to the nearest centimeter. First, we calculated BMI (kg/m^2^) as a continuous variable. Second, we calculated the binary variable normal weight vs. the combined overweight and obesity (OWOB). Thus, we excluded underweight adolescents from the logistic analyses.

We used the Norwegian national reference to calculate age and gender-adjusted BMI, and these values were dichotomized according to the cutoff values for the binary OWOB in the International Obesity Task Force (17, 18). Compared to the Norwegian BMI percentiles, the cutoff for OWOB in 15 to 16-year-old girls follows the 90th percentile. For boys, the cutoff in 15 to 16-year-old boys is located between the 75th−90th percentile (19).

### Exposure Variables

The SDQ was designed as an assessment questionnaire for children's mental health problems (20) and is publicly available (21). The questionnaire can be completed by the adolescents themselves or their parents or teachers, and it is validated internationally as well as in Norway (22–25). We used the self-reported version developed for 11 to 16-year-old children and adolescents (20, 23, 26). It includes 25 items equally divided into five subscales, measuring emotional symptoms, peer problems, conduct problems, hyperactivity, and prosocial behavior. The participants indicated on a three-point Likert scale to which extent a symptom applied to them. Each item was scored 0 for “Not true,” 1 for “Somewhat true,” or 2 for “Certainly true.” The subscale scores range from 0 to 10. On the four subscale scores used in this paper, higher scores indicate more problems. A subscale for internalizing problems (emotional symptoms and peer problems), externalizing problems (conduct problems and hyperactivity), and total difficulties score (externalizing and internalizing problems) can also be calculated (27). SDQ also includes an impact score, which reflects the child's distress and impairment.

In line with our hypothesis, we focused on the four problem subscales. The remaining subscales were beyond our scope.

We scored the SDQ according to the syntax available on the SDQ website (21). We accordingly created subscale scores if at least three out of five items were answered and replaced missing values by mean values.

### Adjusting Variables

We collected information on gender (boy or girl) and age (years and months). We asked if they attended organized leisure-time sports (yes or no) and how often they had breakfast (seldom/never, 1–2 times per week, 3–4 times per week, and 5–6 times per week, daily) and subsequently dichotomized the answers into eating daily breakfast (yes or no) as a proxy for healthy nutrition. We also asked the participants how they perceived their family's economic situation compared to other families (poor, average, good, or very good) as a measure of subjective social status. We used perceived family economy as a categorical variable in the adjusted analyses.

### Statistical Analyses

We estimated the associations between the outcomes BMI (adjusted for age and gender) and OWOB and the four problem subscales of SDQ, conduct problems, hyperactivity, peer problems, and emotional symptoms, using multivariable linear and logistic regression models. We calculated crude and adjusted estimates. Besides, we included effect modifications between genders and the different exposure variables (SDQ subscale score) in order to calculate the gender-specific estimates. The effect modifications term was estimated as a product term. We conducted each multivariable regression analysis separately using the SDQ subscale score as a continuous exposure variable.

Next, we adjusted for clustering on schools by calculating robust standard errors for the 2017 survey only, since data on schools lacked from the 2002 survey. The change in standard errors following adjustment for clustering on school was minimal, which justifies using (cluster-) unadjusted standard errors and *p*-values. We assessed the internal consistency of the SDQ subscale scores using McDonald's Omega and reversed the coding of the items obeys, attends, reflects, friend, and popular prior to this calculation.

Data were analyzed using STATA 15.0 software (STATA, College Station, TX, United States: StataCorp, 2017). McDonald's Omega was calculated using JASP [JASP Team (2019) Version 0.11.1].

## Results

The response rates were 79% in 2002 and 70% in 2017 (Figure 1). The mean BMI was 21.1 kg/m^2^ in 2002 and 21.4 kg/m^2^ in 2017. Compared to 2002, fewer of the adolescents were underweight or normal-weight and more were overweight or obese in 2017 (Table 1).

Table 2 displays the mean SDQ subscale scores stratified by gender for the two time points.

**Table 2:** SDQ scores in Norwegian adolescents by gender in the two surveys from 2002 and 2017.

	**SDQ variable**	***N***	**Mean (*SD*)**	**Range^a^**
**2002**				
Male	Total difficulties	827	8.9 (4.6)	0–28
	Conduct problems	828	2.1 (1.6)	0–10
	Hyperactivity	827	3.6 (2.0)	0–10
	Peer problems	827	1.7 (1.6)	0–8
	Emotional symptoms	828	1.5 (1.7)	0–9
	Prosocial behavior	828	6.8 (1.9)	1–10
	Impact score	822	0.3 (0.9)	0–7
Female	Total difficulties	809	10.3 (5.1)	0–34
	Conduct problems	809	1.9 (1.4)	0–9
	Hyperactivity	809	3.7 (2.0)	0–10
	Peer problems	809	1.4 (1.4)	0–10
	Emotional symptoms	809	3.2 (2.4)	0–10
	Prosocial behavior	809	7.9 (1.6)	0–10
	Impact score	804	0.5 (1.2)	0–10
**2017**				
Male	Total difficulties	743	9.7 (5.2)	0–33
	Conduct problems	743	1.8 (1.6)	0–8
	Hyperactivity	743	4.0 (2.1)	0–10
	Peer problems	743	1.8 (1.7)	0–9
	Emotional symptoms	743	2.1 (1.9)	0–10
	Prosocial behavior	742	7.2 (1.9)	0–10
	Impact score	741	0.3 (1.0)	0–8
Female	Total difficulties	809	11.1 (5.5)	0–36
	Conduct problems	809	1.4 (1.4)	0–9
	Hyperactivity	809	3.7 (2.1)	0–10
	Peer problems	809	1.8 (1.7)	0–9
	Emotional symptoms	809	4.2 (2.6)	0–10
	Prosocial behavior	809	8.0 (1.7)	1–10
	Impact score	806	0.9 (1.7)	0–9

a *Range refers to the minimum and maximum scores in our study sample*.

*SDQ, Strengths and Difficulties Questionnaire*.

**Figure 2:**
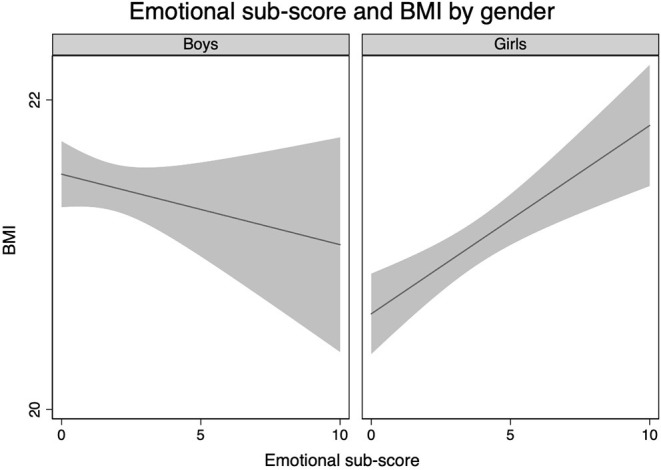
Crude body mass index by the emotional subscale score stratified by gender.

Table 3 displays results from the linear regression analyses. When investigating the association between mental health indices and BMI for boys and girls combined, an increasing BMI was associated with more peer problems (Table 3). Gender modified the associations between emotional symptoms and BMI (Figure 2), between peer problems and BMI (*p* for both effect modifications <0.01), and between conduct problems and BMI [β, −0.14 (95% CI −0.28, −0.02) (*p* for effect modification 0.03)]. There were significant associations between both peer problems and emotional symptoms and increased BMI in girls, and between conduct problems and increased BMI in boys (Table 3). The logistic regression models revealed similar results as the linear models (Table 4).

**Table 3:** Regression coefficients (β) with 95% confidence intervals for the relationship between SDQ subscale scores and BMI in Norwegian adolescents in total and by gender.

**Exposures**	**Boys and girls combined**			**Boys**	**Girls**
**SDQ subscale scores, including total difficulties (sum score) and impact score**	**Crude**	**Adjusted^a^**	**Effect-modification term^b^**	**Adjusted^c^**	**Adjusted^c^**
Total difficulties (*n* = 3,188)	**0.03 (0.01,0.05)**	0.02 (−0.01,0.03)	0.03 (−0.01,0.07)	−0.005 (−0.04,0.03)	0.02 (−0.005,0.05)
Conduct problems (*n* = 3,189)	0.06 (−0.01,0.13)	0.03 (−0.04,0.10)	**−0.14 (−0.28,−0.02)**	**0.10 (0.01,0.19)**	−0.05 (−0.15,0.06)
Hyperactivity (*n* = 3,188)	0.01 (−0.04,0.06)	−0.02 (−0.07,0.04)	−0.03 (−0.13,0.07)	−0.002 (−0.07,0.07)	−0.03 (−0.11,0.04)
Peer problems (*n* = 3,188)	**0.14 (0.07,0.20)**	**0.08 (0.01,0.14)**	**0.20 (0.07,0.33)**	−0.02 (−0.11,0.07)	**0.18 (0.08,0.27)**
Emotional symptoms (*n* = 3,189)	0.03 (−0.02,0.07)	0.02 (−0.03,0.07)	**0.15 (0.05,0.25)**	−0.08 (−0.16,0.002)	**0.07 (0.01,0.13)**
Prosocial behavior (*n* = 3,188)	0.01 (−0.05,0.07)	**0.07 (0.01,0.12)**	0.11 (−0.01,0.23)	0.02 (−0.06,0.10)	**0.12 (0.04,0.21)**
Impact score (*n* = 3,173)	0.07 (−0.01,0.20)	0.03 (−0.05,0.12)	−0.05 (−0.24,0.14)	0.07 (−0.09,0.23)	0.02 (−0.08,0.12)

a *Adjusted for gender, age, year of survey, perceived family economy, member of leisure-time sports team, and eating daily breakfast*.

b *The effect modification term of gender and the exposure variable is adjusted for age, gender, year of survey, perceived family economy, member of leisure-time sports team, and eating daily breakfast*.

c *The exposure variable is adjusted for the effect modification term, gender, age, year of survey, perceived family economy, attended leisure-time sports team, and eating daily breakfast*.

*The n displays number of cases included in the adjusted models, including all participants without any missing information*.

*Numbers in bold indicate statistically significant estimates*.

*BMI, body mass index; SDQ, Strengths and Difficulties Questionnaire*.

**Table 4:** Crude and adjusted odds ratio with 95% confidence intervals for the relationship between SDQ subscale scores and overweight and obesity in reference to normal weight in total and by gender.

**Exposures**	**Boys and girls combined**			**Boys**	**Girls**
**SDQ subscale scores, including total difficulties (sum score) and impact score**	**Crude**	**Adjusted^a^**	**Effect-modification term^b^**	**Adjusted^c^**	**Adjusted^c^**
Total difficulties (*n* = 2,947)	**1.02 (1.01,1.04)**	1.01 (0.99,1.03)	1.03 (0.99,1.07)	1.00 (0.98,1.03)	**1.03 (1.00,1.06)**
Conduct problems (*n* = 2,948)	**1.07 (1.01,1.14)**	1.03 (0.96,1.10)	1.02 (0.89,1.16)	1.02 (0.94,1.11)	1.03 (0.98,1.15)
Hyperactivity (*n* = 2,947)	1.00 (0.96,1.06)	0.98 (0.93,1.03)	0.98 (0.89,1.08)	0.99 (0.93,1.05)	0.96 (0.89,1.04)
Peer problems (*n* = 2,947)	**1.15 (1.09,1.22)**	**1.10 (1.03,1.16)**	**1.13 (1.01,1.27)**	1.04 (0.96,1.13)	**1.17 (1.07,1.28)**
Emotional symptoms (*n* = 2,948)	1.00 (0.97,1.05)	1.02 (0.97,1.07)	**1.11 (1.01,1.22)**	0.96 (0.90,1.03)	**1.07 (1.01,1.13)**
Prosocial behavior (*n* = 2,947)	0.99 (0.94,1.04)	**1.06 (1.00,1.12)**	1.08 (0.95,1.22)	1.04 (0.97,1.11)	**1.12 (1.01,1.24)**
Impact score (*n* = 2,933)	**1.07 (1.00,1.15)**	1.06 (0.97,1.14)	1.06 (0.90,1.25)	1.01 (0.88,1.16)	1.08 (0.98,1.18)

a *Adjusted for gender, age, year of survey, perceived family economy, member of leisure-time sports team, and eating daily breakfast*.

b *The effect modification term of gender and the exposure variable is adjusted for age, gender, year of survey, perceived family economy, member of leisure-time sports team, and eating daily breakfast*.

c *The exposure variable is adjusted for the effect modification term, gender, age, year of survey, perceived family economy, attended leisure-time sports team, and eating daily breakfast*.

*The n displays number of cases included in the adjusted models, including all participants without any missing information*.

*Numbers in bold indicate statistically significant estimates*.

*BMI, body mass index; SDQ, Strengths and Difficulties Questionnaire*.

The statistical models explained little of the total variation in the adolescents' BMI with an *R*^2^ = 0.02.

The McDonald's Omega for the subscale scores was 0.75 for the emotional subscale, 0.59 for the peer subscale, 0.61 for the hyperactivity subscale, 0.52 for the conduct subscale, 0.75 for the impact subscale, and 0.63 for the total difficulties subscale.

The intra-class correlation for schools and BMI in 2017 was 0.007. Adjusting for clustering on schools in 2017 in all full models displayed in Table 3 did not alter any standard errors or *p*-values (data not shown).

Figure 2 depicts the unadjusted association between emotional symptoms and BMI stratified by gender using the lfit-command in STATA.

## Discussion

Our hypotheses were partly supported. We found that higher values of peer problems were associated with higher values of BMI. Furthermore, gender significantly modified the associations. For girls, emotional symptoms, and also peer problems, were associated with an increased BMI, while conduct problems were associated with an increased BMI in boys. Contrary to our hypotheses, we found no association between the hyperactivity subscale and BMI in either of the adjusted analyses. Although the explained variance in BMI was low, these findings can indicate that the association between mental health discomfort and BMI displays gender-related patterns.

We found an association between peer problems and BMI. The association is underpinned by the findings of Strauss and Pollack, who revealed that overweight in adolescence may be a marker of social marginalization and that overweight adolescents had fewer friends compared to normal-weight peers (28). Also, overweight and obese adolescents are at higher risk of being victims of both aggression (29) and relational bullying, including friendship withdrawals (30). OWOB is also found as stigmatizing (31), and thus there is also a possibility that the association is directed from OWOB to peer problems.

We found gender-related patterns in the association between mental health discomfort and BMI. In girls, we found higher subscale scores of both peer problems and emotional symptoms associated with a significantly increased BMI compared to boys. Our finding is in line with previous studies that found gender-related associations between depression and BMI (15, 32, 33) and peer problems and BMI (34). Another study did not find gender-related differences in the association between depression and BMI (12). In boys, we found that those who reported more conduct problems also reported a significantly higher BMI compared to girls. This finding is in line with a previous study reporting an association between behavioral problems and increased BMI in boys (15).

Another important aspect in our found gender-specific pattern in mental health discomfort and BMI can be how boys and girls answer the SDQ. A study found that boys reported more conduct problems and girls reported more emotional symptoms (25). Hence, our study support this gender-specific pattern in reporting symptoms of mental discomfort, and that this is also associated with OWOB.

There is a possibility that the association between mental health discomfort and BMI is mediated through health-related behavior. An underlying factor for health-related behavior, mental health, and obesity is sociodemographic affiliation (35–37). As found in Table 1, only 4% of the adolescents in our study perceived their family economy as poor at both time points. This finding is in line with the egalitarian societies found in Scandinavia. Recent statistics also reveal that only 10% of the total population in Norway have a persistent, low income (38). Thus, results from studies that are affected by sociodemographic affiliation can possibly differ between egalitarian societies and societies with larger differences between sociodemographic groups, as the health-related inequalities are more challenging to uncover in the more egalitarian societies. Still, even if these inequalities are interpreted as minor, further awareness is needed as a recent study found that low sociodemographic status is a risk factor for a cascade of diseases that began with psychiatric disorders as a young adult and were associated with later physical diseases that included OWOB (39). Thus, the known differences in health-related outcomes appear firmly attached to mental health in adolescence and support future research in this area.

Unlike another community-based study (12), we found an association between mental health discomfort and BMI in adolescence. Although the estimates are small, health professionals should be aware of this possible link between mental discomfort and physical health in the general population of adolescents.

As behavior and biology both differ by gender and age during adolescence, the observed gender-related association between mental health discomfort and higher BMI might be different in a sample with a broader age span. Therefore, further studies should explore if the associations are different among older adolescents.

### Strengths and Limitations

We consider the study to have multiple strengths. First, the association between higher BMI and mental health discomfort is found regardless of the 15-year time span and two separate populations. Thus, the association is less likely confounded by small subgroups (12). Second, through the use of SDQ as a well-validated measure of mental health discomfort, we explored both internalizing and externalizing mental health discomfort. The measures of mental health were also not focused on diagnosis. We explored our data according to the well-validated five-factor structure (25). Third, we adjusted the multivariable models for relevant lifestyle variables with well-established associations with BMI. We used daily breakfast as an indicator of a healthy nutritional profile (40) and participation in organized leisure-time sports as a reflection of objectively measured physical activity in accordance with previous studies (41). Fourth, we used a subjective social status to indicate adolescent sociodemographic affiliation, as done by others (42, 43). Subjective social status has been found to reveal distinctive aspects of the social and economic associations besides the objective measures of education, occupation, and income (43) that are not often accomplished by adolescents.

There are some limitations to the current study. First, our data were self-reported. The possibility of random errors due to the self-reported questionnaire contributes to a lower power and an increased chance of type 2 errors. However, results from self-reported SDQ are comparable to the parental-reported SDQ (24), and we consider that self-reporting might have reduced the number of refusals. Second, we did not explore the factor structure of the SDQ, which could have provided information regarding the psychometric results. The poor internal reliabilities for the conduct, hyper, and peer problem subscales might reflect the included negatively worded items, as also found in other studies (44, 45). Thus, the suggested three-factor structure for use in a healthy population could have been approached (27), but this structure is to the best of our knowledge not validated in a Norwegian population. Third, symptom scales such as the SDQ should be interpreted within the cultural frames of the population, and especially regarding behavioral and emotional aspects. Still, more similarities than differences between different populations are found (8). Fourth, the direction of the association between mental health discomfort and obesity is possibly bidirectional (11, 15), but we cannot make such conclusions due to the cross-sectional design.

We suggest that school nurses, practitioners, parents, peers, and researchers should be sensitive to mental health discomfort in adolescents with higher BMI and be aware that the association between mental health and increased BMI may differ for girls and boys. It should be noted, however, that most adolescents with higher BMI in our study did not report mental health discomfort. Furthermore, gender-related approaches in preventing higher BMI may be useful. There is a need for more research on the complex interplay between mental health and weight status in adolescents.

## Conclusion

We found a small association between peer problems and higher BMI in adolescence. We also found that the association between different subscales of the SDQ and BMI was different for boys and girls. This finding indicates that emotional symptoms and peer problems in girls and conduct problems in boys are associated with an increased BMI. Further, there is still a need to explore the gender-related differences in preventive work in adolescent OWOB.

## Data Availability Statement

The datasets generated for this study will not be made publicly available because the data collected in 2002 belong to the Norwegian Public Health Institute, and is licensed for the current study.

## Ethics Statement

The studies involving human participants were reviewed and approved by the Regional Committee for Medical Research Ethics South East approved the studies (2017 Project No. 2016/1755). Written informed consent to participate in this study was provided by participants, and where necessary, the participants' legal guardian/next of kin.

## Author Contributions

AB, TA, and MH-A contributed to the conception and designed of the study. AB and TA planned the data-analysis. AB performed the data analyses. AB, IF, IO, KB, TA, and MH-A interpreted the data, and drafted and completed the manuscript. All authors contributed to the article and approved the submitted version.

## Conflict of Interest

The authors declare that the research was conducted in the absence of any commercial or financial relationships that could be construed as a potential conflict of interest.
